# Bipolar Mood State Reflected in Functional Connectivity of the Hate Circuit: A Resting-State Functional Magnetic Resonance Imaging Study

**DOI:** 10.3389/fpsyt.2020.556126

**Published:** 2020-10-27

**Authors:** Zebin Fan, Jie Yang, Can Zeng, Chang Xi, Guowei Wu, Shuixia Guo, Zhimin Xue, Zhening Liu, Haojuan Tao

**Affiliations:** ^1^Department of Psychiatry, The Second Xiangya Hospital, Central South University, Changsha, China; ^2^National Clinical Research Center for Mental Disorders, Changsha, China; ^3^National Technology Institute on Mental Disorders, Changsha, China; ^4^Mathematics and Computer Science College, Hunan Normal University, Changsha, China

**Keywords:** bipolar disorder, mood states, hate circuit, resting-state fMRI, functional connectivity

## Abstract

**Background:**

Previous studies suggested bipolar disorder caused an aberrant alteration in the insular, putamen, and left superior frontal gyrus, which are the main components of the hate circuit. However, the relationship between the hate circuit and the pathophysiologic substrate underlying different phases of bipolar disorder remain unclear. In this study, we aimed to identify group differences of resting-state functional connectivity within the hate circuit in healthy controls (HCs) and bipolar patients in different mood states.

**Methods:**

Resting-state functional magnetic resonance imaging of the brain were acquired from 54 HCs and 81 patients with bipolar disorder including 20 with bipolar mania (BM), 35 with bipolar depression (BD), and 26 with bipolar euthymia (BE). We selected bilateral insula (L.INS and R.INS), bilateral putamen (L.PUT and R.PUT), and left superior frontal gyrus (L.SFGd) as seed regions, and conducted the seed-based functional connectivity analysis to identify group differences of connectivity strength within the hate circuit. Spearman correlations were performed to evaluate the relationship between the hate circuit and manic/depressive symptoms.

**Results:**

Significant group differences of connectivity strength within the hate circuit were found in links of the R.INS-L.SFGd, R.PUT-L.SFGd, and L.INS- R.PUT after false discovery rate was corrected. The BM group showed an opposite hate circuit pattern to BD, BE, and HCs. The BD group showed decreased hate circuit connectivity in the L.INS-R.PUT compared with the BE group. No significant difference was detected among BD, BE, and HCs. Furthermore, functional connectivity of the R.INS-L.SFGd and R.PUT-L.SFGd were positively correlated with manic symptoms, while the L.INS- R.PUT was negatively correlated with depressive symptoms.

**Conclusions:**

Our preliminary findings suggest that altered functional connectivity of the hate circuit in different mood phases may be related to state markers and underpin the neuropathological basis of bipolar disorder.

## Introduction

Bipolar disorder is a severe mental disorder with a high risk for suicide-related events (1) and has become a leading cause of global disease burden. Bipolar disorder is characterized by mood instability with alternating periods of depression (bipolar depression, BD), (hypo) mania (bipolar mania, BM), and euthymic mood states (bipolar euthymia, BE) (2) and is associated with brain functional dysconnectivity (3). Many findings from structural and functional magnetic resonance imaging (fMRI) studies in bipolar patients have indicated abnormalities in the frontal-limbic pathway, which is responsible for emotional regulation and cognitive control (4–10), However, to date, the pathophysiologic substrate underlying different phases of bipolar disorder remain underexplored (11, 12).

Several studies have concurrently focused on the distinctions of brain function across different mood states in bipolar disorder and found some state-related changes in BM and BD and trait-related neural abnormalities in BE (3, 10, 13–21). Specifically, functional imaging showed hypometabolism within the frontal cortex occurring in depression, while hypermetabolism in mania was observed (21, 22). During cognitive or emotional tasks, the reduced activation in the prefrontal cortex may suggest a trait abnormality across all mood states. Furthermore, the direction of signal changes in the prefrontal gyrus and the subcortical brain regions involving the insula, putamen, and other subcortical areas may be associated with specific acute mood states (10, 18–20). Notably, Hummer TA and colleagues explored brain activation in bipolar disorder across the three different mood phases in an fMRI study using emotional response inhibition tasks, and they found distinct trait- and state-related neural abnormalities mainly associated with the superior frontal gyrus, insula, and putamen in bipolar disorder (10). We noted that the location of the abnormally activated brain regions in the above studies almost overlapped with the main components of the so-called ‘hate circuit’, which is activated when individuals view people who they hate (23).

Hate is one of the intense basic human emotions and plays an important role in psychological behavior and human evolution (24); its core feeling of disgust has been implicated in a wide range of psychological and psychiatric conditions (25–30). Of particular note is that an abundance of research has suggested that hate is critically associated with depressive symptoms (29, 30). More intriguingly, in our previous study, uncoupling of the ‘hate circuit’ was detected in both first-episode and longer-term depressive patients (31). The depressive phase of bipolar disorder is broadly similar to unipolar depression in clinical manifestation, which often obscures the diagnostic distinction. This might hint at the overlap in the neuropathology of depression (32). Furthermore, mania and depression are clinically polar opposites of mood. Thus, it is valuable for us to consider whether the hate circuit is also uncoupled in BD. If so, will the functional connectivity within the hate circuit be enhanced in BM with contrary symptoms to depression? However, to our knowledge, the hate circuit has not been studied in bipolar disorder. Thus, it could be instructive to examine the connectivity pattern of the hate circuit in the different mood states for clues into the trait-related and state-related mark of bipolar disorder, and therefore provide assistance for finding a promising novel biomarker of diagnosis and possible clinical intervention.

In this study, we explored the functional connectivity of the hate circuit underlying different phases of bipolar disorder. Considering the opposing polar of mood and cognitive and psychomotor symptoms in the different bipolar phases, it is possible to hypothesize that functional connectivity of the hate circuit may be different between BM and BE. To test this hypothesis, seed-based analysis in resting-state fMRI was adopted to examine correlations in spontaneous fluctuations among regions of interest (ROIs) in patients with bipolar disorder across different mood states and HCs. Resting-state fMRI is a non-invasive technique that occurrs in the absence of any explicit task or stimulus which can provide measures of tonic functional connectivity in neural circuits of interest and avoid some limitations of the task-related fMRI (33–35). We chose the bilateral insula (i.e., L.INS and R.INS), bilateral putamen (i.e., L.PUT and R.PUT), and left superior frontal gyrus (dorsolateral part; L. SFGd) as ROIs according to the main abnormal connectivity in depression of our prior study (31). Then, group differences of the functional connectivity strength within the hate circuit were detected using a one-way analysis of covariance (ANCOVA). Finally, the relationship between depressive/manic symptoms and the altered functional connectivity strength were calculated by Spearman correlation.

## Materials and Methods

All study procedures were approved by the medical ethics committee of the Second Xiangya Hospital, Central South University. Prior to obtaining consent, the capacity to provide informed consent for all potential participants was ascertained by two licensed psychiatrists. After explaining the study procedures, informed written consent was obtained from all participants. All study procedures were conducted in strict accordance with the Declaration of Helsinki.

### Study Sample

In this study, a total of 91 patients with bipolar disorder and 54 HCs were recruited from the inpatient or outpatient department of the Second Xiangya Hospital, Central South University. To minimize the effects of age on brain function and to ensure adequate understanding and expressive capacity, only right-handed Han Chinese aged 18 to 45 years who had at least 9 years of formal education were recruited.

Considering the possible heterogeneity of bipolar disorder type I and bipolar disorder type II (36, 37), only patients with bipolar disorder type I were included in our present study. Patients diagnosed with bipolar disorder type I (bipolar depression, bipolar mania, or euthymic bipolar disorder) were assessed face to face by two experienced psychiatrists using the Structured Clinical Interview for DSM-IV Axis I Disorders, Patient Edition (SCID-I/P) (38). Depressive and manic symptoms were assessed face to face by a psychiatrist using the 17-item version of the Hamilton Depression Rating Scale (HDRS_17_) (39) and the Young Mania Rating Scale (YMRS), respectively (40). In our present study the YMRS and HDRS_17_ were completed by an experienced psychiatrist based on the structured interview guide. The scores were based on patients’ self-reports combined with clinician’s observations in the past week. For patients with BD, the HDRS_17_ total score should be at least 17 and YMRS total score should be less than 6; for patients with BM, the YMRS score was above 12 and the HDRS_17_ score less than 8; for patients with BE, the HDRS_17_ score should be no more than 8 and the YMRS score should be less than 6.

HCs were evaluated face to face by a psychiatrist using the Structured Clinical Interview for DSM-IV, Non-Patient Edition (SCID-I/NP) (41). All HCs had no lifetime history of any psychiatric disorders and no first-degree relatives with a history of psychiatric disorders, and the HCs group was age and gender-matched with all three patient groups.

Participants were excluded if they had any of the following: (1) History of neurological diseases or other serious physical diseases; (2) History of electroconvulsive therapy; (3) History of drugs, alcohol, and other psychoactive substance abuse; (4) Comorbidities with other Axis I or Axis II disorders; (5) Any contraindications for MRI; and (6) Had drunk alcohol or taken benzodiazepines within 24 hours prior to the interview and fMRI scanning.

### fMRI Data Acquisition and Preprocessing

Imaging data were collected using a 3.0 Tesla Philips Gyrosan Achieva (Amsterdam, The Netherlands) scanner. Participants were explicitly instructed to lie quietly with their eyes closed and stay still in the scanner. Participants were requested to clear their minds to the best of their ability without falling asleep (resting state). A gradient-echo echo-planar imaging (EPI) sequence was used with the following parameters: Repeat time =2000ms, Echo time=30ms, Field of view (FOV) =240mm×240mm, Flip Angle = 90°, Matrix = 64×64, Voxel size =3mm×3mm×3mm, axial slice= 36, slice thickness= 4mm, scanning interval = 0mm, 250 time points. The total scan time was 500s for each participant.

Before fMRI data preprocessing, the first ten volumes were removed to allow for magnetization equilibrium and the subjects’ adaptation to the environment (42). Resting-state fMRI data preprocessing was conducted by DPABI (43). The remaining 240 functional scans were first corrected for within-scan acquisition time differences between slices and realigned to the middle volume to correct for inter-scan movements. All participants in this study had less than 3 mm maximum displacement in x, y, or z and less than 3° of angular rotation about each axis. Then the functional scans were spatially normalized to the standard template (Montreal Neurological Institute), resampled to the voxel size of 3×3×3 mm^3^, and smoothed with a Gaussian kernel of 4×4×4 mm^3^ full-width at half maximum. Subsequently, the BOLD signal of each voxel was firstly detrended to abandon linear trends and then passed through a band-pass filter (0.01-0.08 Hz) to reduce low-frequency drift and high-frequency physiological noise. Finally, nuisance covariates, including head motion parameters, global mean signals, white matter signals, and cerebrospinal fluid signals, were regressed out from the BOLD signals. In line with our prior studies (44, 45), we controlled for head motion first through regression of six head motion parameters plus their temporal first derivatives. We also used scrubbing by removing outlier volumes, defined as frame-wise displacement (FD) of more than 0.5 mm from the previous frame or global mean intensity of more than 2 SDs. If more than 48 volumes were scrubbed (ie, >20% of the acquired volumes), we excluded these subjects from subsequent analysis. Ten patients were excluded because of excessive head movement during fMRI scanning. Therefore, there were eighty-one patients involved in the present research analysis with 20 BM patients, 35 BD patients, and 26 BE patients. Details of current psychotropic medication are provided in **Supplementary Table S1**.

### Definition of Seed Regions and Functional Connectivity

We selected the five seed regions (L.SFGd, L.INS, R.INS, L.PUT, and R.PUT) as ROIs based on the reasons mentioned in the introduction. In the present study, ROIs were generated from an automated anatomical labeling (AAL) template which was employed to segment the brain into 90 regions of interest in previous studies (31, 46).

For each ROI, the BOLD time series of the voxels within it was averaged to generate the reference time series for this region. For each subject and each ROI, a functional connectivity correlation matrix was produced by computing the correlation coefficients between the reference time series of one ROI and it from every other ROI. Therefore, a 5×5 symmetric connection matrix and ten links between all pairs of ROIs were obtained for each participant. Functional connectivity correlation coefficients were converted to z values using Fisher’s r-to-z transform (z-FC) to improve the normality. Therefore, each z-FC represented the strength of the functional connectivity in every pair of ROIs.

### Statistical Analysis

All the general demographic and clinical assessment data and z-FC values were fed into the Statistic Package for Social Science (SPSS) 22.0 (SPSS, Inc., Chicago, IL). First, one-way ANOVA was performed to compare the group differences in age, education, duration of illness, HDRS_17_ total score, and YMRS total score. A Chi-square test was used to compare the gender difference among the four groups. We then performed a one-way analysis of covariance (ANCOVA, two-tailed) comparing zFC values for all ROI pairs in the 5×5 matrix for the four groups (BM, BD, BE, and HC). Next, we corrected the resulting p-values for multiple comparisons using a false discovery rate (FDR) correction to control Type I errors at the threshold with p < 0.05. Post-Hoc testing for differences between individual groups was performed using the least significant difference (LSD) test. Finally, Spearman correlation analysis was used to evaluate the relationship between the altered functional connectivity and the HDRS_17_ total score and YMRS total score.

## Results

### Demographic and Clinical Characteristics

Demographic information and clinical characteristics were summarized in **Table 1**. There were no significant differences in age (ANOVA, F = 0.610, p = 0.610) or gender (χ2 = 1.262, p=0.738). The patient groups were matched with duration of illness (ANOVA, F = 1.475, p = 0.235) and Chlorpromazine equivalents (ANOVA, F = 3.045, p = 0.053). However, the BM group had a lower education level than the BD, BE, and HCs group (ANOVA, F = 3.150, p = 0.027; LSD, p<0.05). The BD group had a significantly higher HDRS_17_ score than BM and BE groups, while the BM group had a higher YMRS score than the BD and BE groups. Detailed results of Post-hoc LSD tests in education level, HDRS_17_ score, and YMRS score were also provided in **Table 1**.

**Table 1:** Demographic and clinical profiles of patients with BM, BD, BE, and HC.

Characteristics	BMn = 20	BDn = 35	BEn = 26	HCn = 54	Analysis	
(mean ± SD)					F/χ2	*p*
Age (y)	27.80 ± 8.10	26.37 ± 6.20	26.54 ± 6.81	25.48 ± 6.44	0.610	0.610
Gender (F/M)	12:8	20:15	13:13	34:20	1.262	0.738
Education (y)	10.95 ± 2.78	13.04 ± 3.07	13.12 ± 2.69	12.91 ± 2.83	3.150	0.027*^a^
Duration of illness (m)	81.92 ± 89.19	54.43 ± 51.47	54.00 ± 50.54	N/A	1.475	0.235
Chlorpromazine equivalents (mg)	202.08 ± 140.59	112.40 ± 144.12	221.79 ± 250.01	N/A	3.045	0.053
HDRS_17_	2.85 ± 2.41	20.97 ± 4.58	3.04± 2.14	N/A	268.08	<0.001*^b^
YRMS	23.00 ± 7.03	1.69 ± 1.89	1.50 ± 2.00	N/A	231.77	<0.001*^c^

BM, bipolar mania; BD, bipolar depression; BE, euthymic bipolar disorder; HC, healthy controls; HDRS_17_, 17 item-Hamilton Depression Rating Scale; YRMS, Young Mania Rating Scale.

*ANOVA, p < 0.05 (two tailed). ^a^LSD, p < 0.05 (two tailed). BM<BD, BE, HC; ^b^LSD, p < 0.05 (two tailed). BD > BM, BE; ^c^LSD, p < 0.05 (two tailed). BM>BD, BE.

### Abnormal Functional Connectivity in Hate Circuit

**Figure 1:**
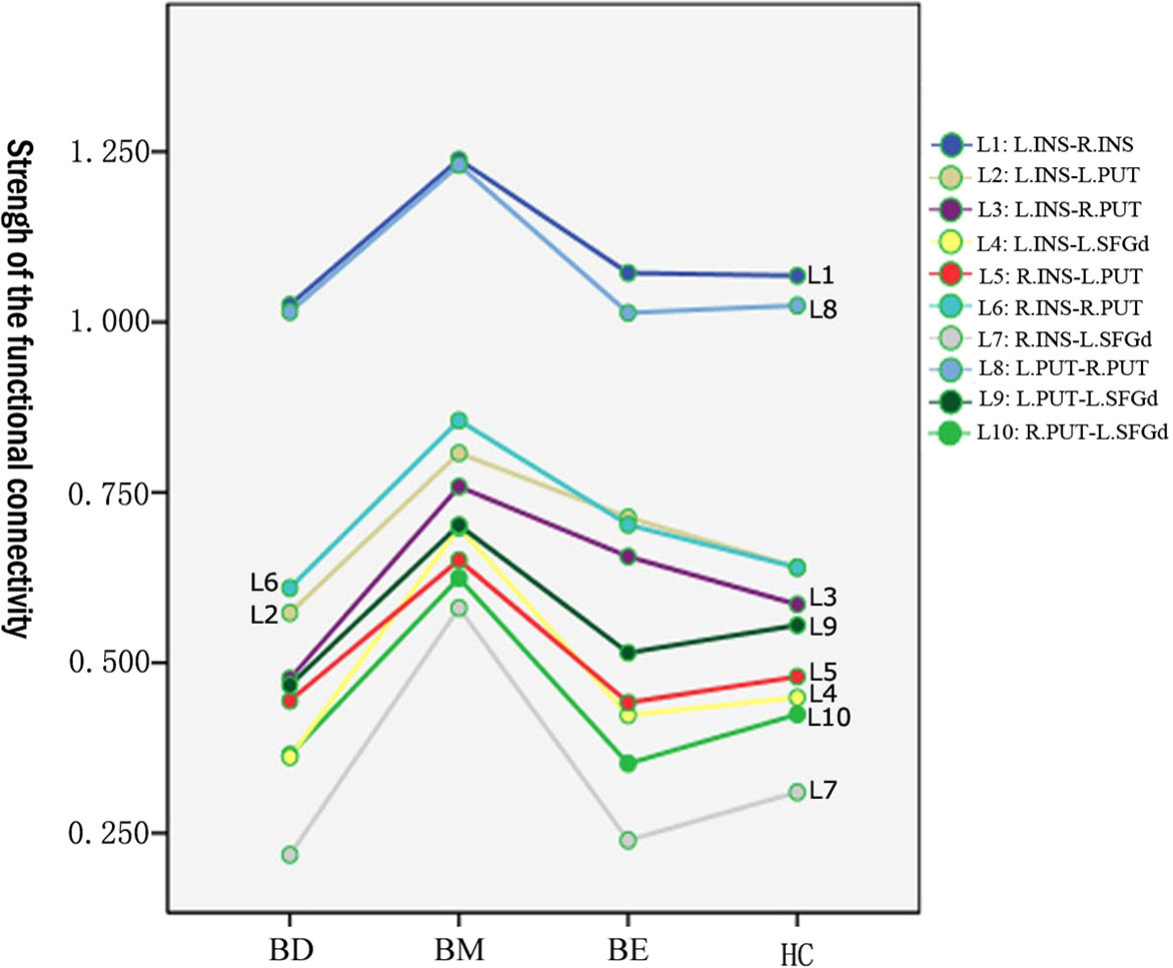
Means value of the functional connectivity strength of each link in the hate circuit in BM, BD, BE, and HC groups. BD, bipolar depression; BM, bipolar mania; BE, euthymic bipolar disorder; HC, healthy controls.

The mean value of each functional connection in the four groups were shown in **Table 2**. A trend of higher mean values of the functional connectivity strength within the hate circuit was shown in the BM group while a lower trend was shown in the BD group relative to the HCs group. The mean value of the functional connectivity strength within the hate circuit in the BE group was comparable to that of the HCs group (see **Table 2** and **Figure 1**).

**Table 2:** Mean value of functional connectivity in each group and the group difference analysis.

Links(mean ± SD)	BM	BD	BE	HC	ANCOVA		
					F	*p* (FDR uncorrected	*p* (FDR corrected)
L.INS-R.INS	1.22 ± 0.48	1.03 ± 0.38	1.08 ± 0.32	1.07 ± 0.34	1.343	0.263	0.263
L.INS-L.PUT	0.79 ± 0.52	0.58 ± 0.33	0.72 ± 0.28	0.64 ± 0.29	2.105	0.103	0.124
L.INS-R.PUT	0.74± 0.37	0.48 ± 0.24	0.66 ± 0.27	0.59 ± 0.30	4.531	0.005	0.025*
L.INS-L.SFGd	0.68 ± 0.46	0.37 ± 0.35	0.43 ± 0.35	0.45 ± 0.34	3.374	0.020	0.05
R.INS-L.PUT	0.63 ± 0.46	0.45 ± 0.28	0.47 ± 0.28	0.48 ± 0.30	2.380	0.073	0.109
R.INS-R.PUT	0.84 ± 0.53	0.61 ± 0.31	0.71 ± 0.26	0.64 ± 0.29	2.771	0.044	0.088
R.INS-L.SFGd	0.56 ± 0.54	0.22 ± 0.27	0.24 ± 0.31	0.31 ± 0.29	5.175	0.002	0.020*
L.PUT-R.PUT	1.21 ± 0.35	1.02 ± 0.33	1.02 ± 0.30	1.03 ± 0.31	2.034	0.112	0.124
L.PUT-L.SFGd	0.68 ± 0.47	0.47 ± 0.27	0.52 ± 0.29	0.58 ± 0.24	2.342	0.076	0.109
R.PUT-L.SFGd	0.60 ± 0.45	0.37 ± 0.23	0.36 ± 0.26	0.43 ± 0.25	3.872	0.011	0.037*

L, left; R, right; BM, bipolar mania; BD, bipolar depression; BE, euthymic bipolar disorder; HC, healthy controls; SFGd, superior frontal gyrus (dorsolateral); INS, insula; PUT, putamen.

*FDR corrected, p < 0.05.

After adjusting for education level and FDR correction, significant group differences of functional connectivity strength were found in L.INS-R.PUT (p=0.005; FDR corrected p=0.025), R.INS-L.SFGd (p=0.002; FDR corrected p=0.02), and R.PUT-L.SFGd (p=0.011; FDR corrected p=0.037) (see **Table 2**). A significant trend of group differences was shown in L.INS-L.SFGd (p=0.02; FDR corrected p=0.05).

**Figure 2:**
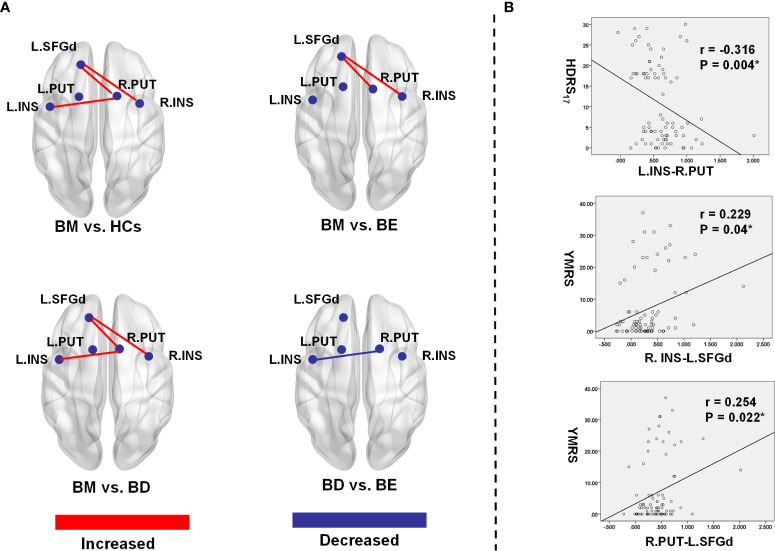
Significant group differences of functional connectivity strength in the hate circuit. **(A)** The spatial location of the altered functional connectivity in the hate circuit; **(B)** The correlation between the altered functional connectivity and clinical symptoms. L, left; R, right; BM, bipolar mania; BD, bipolar depression; BE, euthymic bipolar disorder; HCs, healthy controls; SFGd, superior frontal gyrus (dorsal); INS, insula right PUT, putamen; HDRS_17_, 17 item-Hamilton Depression Rating Scale; YRMS, Young Mania Rating Scale.

Post-hoc LSD showed that the functional connectivity strength of the R.INS-L.SFGd and R.PUT- L.SFGd in the BM group were significantly higher than that in the BD, BE, and HCs groups. However, there were no significant differences in the functional connection values of the R.INS-L.SFGd and R.PUT- L.SFGd among BD, BE, and HCs. Moreover, the functional connectivity of the L.INS-R.PUT in the BD group was significantly lower than that in BM and BE groups, while in the BM group it was higher than HCs. No significant difference in the functional connectivity strength of L.INS-R.PUT was detected between BM and BE or among BD, BE, and HCs (see **Table 3** and **Figure 2**).

**Table 3:** Between-group differences of the significant changed links in the hate circuit (p-values for LSD post-hoc testing).

Links	Post-hoc LSD p value					
	BM vs. BD	BM vs. BE	BM vs. HC	BD vs.BE	BD vs. HC	BE vs. HC
L.INS-R.PUT	0.001*	0.206	0.021*	0.019*	0.084	0.319
R.INS-L.SFGd	<0.001*	0.001*	0.003*	0.808	0.212	0.383
R.PUT-L.SFGd	0.003*	0.003*	0.013*	0.865	0.341	0.294

L, left; R, right; BM, bipolar mania; BD, bipolar depression; BE, euthymic bipolar disorder; HCs, healthy controls; SFGd, superior frontal gyrus (dorsolateral); INS, insula; PUT, putamen.

*p < 0.05.

### Correlations Between Abnormal Functional Connectivity and Clinical Variables

In our present study, a significant positive Spearman correlation was found between the YMRS score and the functional connectivity of the R.INS-L.SFGd (r=0.229, p=0.04) and R.PUT-L.SFGd (r=0.254, p=0.022), while a significant negative correlation was detected between the HDRS_17_ score and functional connectivity strength of L.INS-R.PUT (r=-0.316, p=0.004) (please see **Table 4** and **Figure 2**).

**Table 4:** Spearman correlation analysis between clinical symptoms and the functional connectivity of each significant different link in the hate circuit among the four groups (p<0.05, two tailed).

Variable		L.INS-R.PUT	R. INS-L.SFGd	R.PUT-L.SFGd
HDRS_17_	r	-0.316	-0.108	-0.055
	*p*	0.004*	0.337	0.624
YMRS	r	0.190	0.229	0.254
	*p*	0.090	0.040*	0.022*

L, left; R, right; INS, Insula; PUT, Putamen; SFGd, Superior frontal gyrus (dorsolateral); HDRS_17_, 17 item-Hamilton Depression Rating Scale; YRMS, Young Mania Rating Scale.

* p<0.05, two tailed.

## Discussion

In the present study, we found that the functional connectivity pattern of the hate circuit showed significant discrepancies in different mood phases of bipolar disorder. Specifically, the ordinal ranking of the R.INS-L.SFGd, R.PUT-L.SFGd, and L.INS-R.PUT strengths were: BM>HCs ≈ BE>BD. Functional connectivity strengths of the R.INS-L.SFGd, R.PUT-L.SFGd, and L.INS-R.PUT were significantly increased in BM compared with BD, BE, and HCs, whereas the opposite connectivity pattern was observed in BD compared with BM and BE. Additionally, the connectivity pattern of the hate circuit in BE was similar to HCs. Furthermore, connectivity strengths of the R.INS-L.SFGd and R.PUT-L.SFGd positively correlated with scores of the manic symptoms while those of the L.INS-R.PUT negatively correlated with scores of depressive symptoms.

In our present study, the functional connectivity pattern of the hate circuit was capable of distinguishing the BM from BD, BE, and HCs, and could also distinguish the BD from BE. The diverse functional connectivity pattern of the hate circuit in different mood phases suggested state-related markers of bipolar disorder. The relationships between the connectivity strength of R.INS-L.SFGd, R.PUT-L.SFGd, and manic symptoms and relationship between L.INS-R.PUT and depressive symptoms were further supported by our significant correlation findings. Increased functional connectivity of the R.INS-L.SFGd and R.PUT-L.SFGd were positively correlated with the YMRS score while decreased functional connectivity of the L.INS-R.PUT was negatively correlated with the HDRS_17._ These findings suggested that mania and depression were associated closely with the hate circuit.

Moreover, the altered functional connectivity in the insula, putamen, and lateral prefrontal gyrus in BM patients provides neuropathological evidence for the impaired emotional regulation and cognitive control in bipolar disorder. In anatomy, the insula cortex reciprocally connects with the frontal lobe and projects to parts of the brain that involve the putamen (47). In terms of brain function, the lateral superior frontal gyrus plays a critical role in mental manipulation and information monitoring (48, 49). The insula is recognized as an integration hub implicated in cognitive, emotional, motor, and somatosensory activity (50–52) and the right putamen is considered to be closely associated with motor control, reinforcement learning, and semantic processes. Therefore, this neural pathway was considered to be responsible for emotional regulation and cognitive control (48, 49). Moreover, higher activation in the right insula, right putamen, and left lateral prefrontal cortex had been found in BD and BM patients during the response to inhibiting sad faces compared with HCs (10). Both mania and depression patients showed reduced dorsolateral prefrontal activation during an N-back working memory task, although this may improve partially in euthymia (53).

Accordingly, we postulated that the hate circuit may play a critical role in the pathophysiologic substrate of mania and depression. It was proposed that the uncoupling hate circuit in depression might impair cognitive control over feelings of hate toward the self or others during social interactions. This could result in improper feelings of hate and therefore increase uncontrolled self-loathing and withdrawal from social interactions (31). Considering the opposite symptoms in depression and mania, we conjectured that increased functional connectivity in hate circuit could also undermine the emotional regulation of hate during social interactions and may later lead to the reversed symptoms of depression (manic episode) such as euphoria, pathological narcissism, impulsivity, and overactive social interactions. Taken together, our findings not only support the association between decreased connectivity of the hate circuit with depression but also add new evidence that supports the links between hate circuit and mania.

The opposite functional connectivity patterns in BM and BD found in our study are in line with a growing body of neuroimaging literature, although abnormal connectivity may locate in different circuits. For example, right amygdala-hippocampal connectivity was increased in BM but decreased in BD (54). BM patients showed contrasting variability patterns in the default mode network (DMN) and sensorimotor networks (SMN) balance to BD patients (55). The contrasting functional connectivity pattern of different neural circuits in bipolar disorder may reflect the “spatiotemporal psychopathology” that was proposed by Northoff G (56) and suggests dynamic instabilities of large-scale brain networks in bipolar disorder (57). Notably, Altinay et al. found BD patients showed increased connectivity between the left dorsal caudal putamen and right superior temporal gyrus extending to the insula compared with both BM and HCs using seed-based analysis to measure differences in resting-state functional connectivity of six striatal sub-regions (58). However, in our study, no significantly different functional connectivity between the left putamen and right insula were found. Brady RO Jr and colleagues used unbiased whole-brain analyses to measure spontaneous brain activity in patients with bipolar disorder in mania and euthymia, and they found that within the dorsal attention network, the mania group demonstrated hyper-connectivity while the bipolar euthymia group showed hypo-connectivity compared with HCs. When the bilateral frontal lobe was selected as an ROI, the results still corresponded to the findings of whole-brain analyses (14). Thus, further studies investigating cognitive testing and emotional processing during MRI scanning and performing large-scale brain network analysis are needed to ascertain the role different neural circuits play and how they interact with each other in the psychopathology of medication-free bipolar disorders.

We found decreased functional connectivity of the L.INS-R.PUT in BD patients compared to BE patients and a decreased tendency of functional connectivity strength within the hate circuit in BD patients relative to HCs. However, contrary to our hypothesis, a significant functional connectivity difference of the hate circuit was not detected in BD compared with HCs. Although in line with some studies that found no significant different brain activation in BD compared with HCs (19), the majority of resting-state functional magnetic resonance imaging studies had reported altered fronto-limbic functional connectivity in major depressive disorder or BD patient groups compared HCs (54, 59, 60). Altinay MI and colleagues found that the BD patients demonstrated increased functional connectivity between the left putamen and frontal gyrus, as well as increased connectivity of the right putamen with the right superior temporal gyrus extending into the insula compared with HCs (58). Kandilarova S and colleagues found significantly reduced effective connectivity of the anterior insula to the middle frontal gyrus in the depressive group compared to the healthy subjects (59). Decreased functional connectivity between the dorsolateral prefrontal cortex and insula was also found in subjects with subthreshold depression compared to healthy controls (61). However, sample heterogeneity, different analytical methods, and medication effects may have contributed to the inconsistent findings. As for our negative findings, another probability is that decreased functional connectivity in the hate circuit might appear only in unipolar depression but not in bipolar depression. If confirmed in a larger sample size, then the functional connectivity pattern of the hate circuit could be a potential diagnostic biomarker for unipolar depression and bipolar depression. However, further studies with a relatively large sample size are needed to verify our assumption.

Consistent with our hypotheses, the connectivity pattern of the hate circuit in BE patients was not different from that of HCs in the current study. Our results are in line with many previous studies that showed negative findings using whole-brain independent component analysis (ICA), seed-based analysis, or other topographical patterns analysis to explore resting-state functional connectivity in patients during periods of euthymia (55, 62, 63). For instance, the absence of hyper- or hypo-connectivity in the default mode network, frontoparietal network, and salience network had been demonstrated in euthymic bipolar patients in resting-state functional connectivity studies (62). Patients in the euthymic phase did not show any significant differences in the Slow5 fractional SD DMN/SMN ratio compared with HCs (55). Moreover, Syan SK and colleagues used the dorsolateral prefrontal cortex (dlPFC) as a primary ROI in euthymic women but no aberrant functional connectivity was detected between dlPFC and the insula or putamen, although there was increased resting-state functional connectivity between the dlPFC and the brainstem (63). Furthermore, these studies suggested that medication may have treatment effects on normalizing the dysfunctional neural circuit, since patients were relieved from the acute disease phase after a period of antipsychotic medication. The normalization of brain networks in patients during the euthymia phase may inform the neurobiology and compensatory brain mechanisms maintaining clinical remission (62). However, findings from seed-based analysis studies remain inconsistent. Aberrant resting-state functional connectivity with the prefrontal cortex, amygdala, cingulate cortex, and somatosensory cortex were reported in studies using seed-based analysis in patients during euthymic phase compared with HCs. The resting-state functional connectivity changes in the euthymic phase may reflect the trait-based pathophysiology of bipolar disorder (62, 64).

The present study has several limitations. First, this is a cross-sectional study which can only observe the current time measurements of the brain’s functional connectivity. The bipolar patients were not longitudinal follow-up subjects across mood states. Second, almost all patients were taking medication(s) at the time of the scan, so the potential effects of medication in the present study must be considered. Third, relatively small samples may limit statistical power, especially the small sample size in the BM group. Finally, although the seed-based analysis adopted in this study is a hypothesis-driven approach that can directly answer a direct question, it may disregard the richness of information available within the statistical relationships between multiple data points and, furthermore, the selecting of seed may bias connectivity findings towards specific, smaller neural circuits rather than large-scale brain networks (65). Thus, any generalizations about the findings need to be performed with caution. Further longitudinal studies with larger samples of medication-free subjects will be necessary to verify the altered functional connectivity patterns of the hate circuit across different mood phases at the large-scale brain network level.

## Conclusions

In summary, we explored group differences of resting-state functional connectivity within the hate circuit in HCs and patients with bipolar mania, bipolar depression, and bipolar euthymia using seed-based functional connectivity analysis. We found that the functional connectivity strength within the hate circuit was distinctive in different mood phases of bipolar disorder. Patients with BM showed increased hate circuit compared with all other groups, while patients with BD showed decreased hate circuit compared with BM and BE. Additionally, patients with BE had a similar connectivity pattern of the hate circuit to HCs. Furthermore, the severity of manic and depressive symptoms was significantly correlated with connectivity strength of the hate circuit. We speculated that increased hate circuit might be underlying the neuropathology of BM while decreased hate circuit might be underlying the neuropathology of BD. The altered functional connectivity of the hate circuit in different mood phases might be state-related markers of bipolar disorder. Our findings may shed a novel light on the neuropathology of bipolar disorder. If confirmed in a larger sample size, it may carry important implications for the understanding of a potential switching mechanism of different mood states and provide new targets for treatment.

## Data Availability Statement

The datasets presented in this article are not readily available because ethical restrictions. Requests to access the datasets should be directed to Data are available upon reasonable request to ZL, zningl@163.com.

## Ethics Statement

The studies involving human participants were reviewed and approved by Medical Ethics committee of the second Xiangya Hospital, Central South University. The patients/participants provided their written informed consent to participate in this study.

## Author Contributions

Data collection: ZF, CZ, GW, CX. Methodology: JY, ZF, HT. Writing (original draft preparation): ZF, Writing (review and editing): ZF, JY, CZ, CX, GW, SG, ZX, ZL, HT. Project administration: HT, ZL. All authors contributed to the article and approved the submitted version.

## Funding

This study was supported by the National Natural Science Foundation of China (grant numbers 81301161, 81701325, 82071506, 81801353 and 81671335), the China Precision Medicine Initiative (grant number 2016YFC0906300), and the Natural Science Foundation of Hunan Province, China (grant numbers 2019JJ50848).

## Conflict of Interest

The authors declare that the research was conducted in the absence of any commercial or financial relationships that could be construed as a potential conflict of interest.
