# Supplementary material for: Bipolar Mood State Reflected in Functional Connectivity of the Hate Circuit: A Resting-State Functional Magnetic Resonance Imaging Study
Source: Front Psychiatry. 2020 Oct 27;11:556126. doi: 10.3389/fpsyt.2020.556126 (PMC7652934; doi:10.3389/fpsyt.2020.556126)
Supplement: Supplementary file 1 [file Table_1.pdf]

Bipolar Mood State Reflected in Functional Connectivity of hate circuit: A Resting-State functional magnetic resonance imaging study

Supplemental Information

Supplemental Methods & Materials

Current psychotropic medication of participants

Here briefly describe the psychotropic treatment of all participants. Seventy-six bipolar patients (93.8%) were taking medications. There were 55 patients (67.9%) taking mood-stabilizers (including lithium, valproate and lamotrigine, 37 patients on lithium) and 60 patients (74.1%) were taking antipsychotics (all were atypical antipsychotics including clozapine, quetiapine, risperidone and olanzapine), and 47 patients (58.0%) were taking two types of medications mentioned above. Tween-seven patients (33.3%) were on antidepressants and all of them were in depression or euthymic phase. Five depressive patients (6.2%) were using benzodiazepines (no taking benzodiazepines within 24 hours prior to the interview and fMRI scanning). Five patients (6.2%) were medication-free. For detailed information of psychiatric medications patients used in each group, please see Table S1.

Table S1. Psychiatric medications patients used in each group

	**BM** n=20	**BD** n=35	**BE** n=26	**Total**
Medication	19（23.5%）	31（38.3%)	26(32.1%)	76(93.8%)
Medication-free	1（1.2%）	4（4.9%）	0（0.0%）	5（6.2%）
Mood-stabilizers	17(21.0%)	14(17.3%）	24（29.6%）	55（67.9%）
Lithium	13（16.0%)	8(9.9%)	16（19.8%）	37（45.7%）
Antipsychotics	18（22.2%）	21（25.9%）	21（25.9%）	60（74.1%）
Antidepressants	0（0%）	20（24.7%）	7（8.6%）	27（33.3%）
Antipsychotics and Mood-stabilizers	16（19.8%）	13（16.0%）	18（22.2%）	47（58.0%）
Benzodiazepines	0（0.0%）	5（6.2%）	0（0.0%）	5（6.2%）
